# Ultrasound Imaging in Psoriatic Arthritis: What Have We Learnt in the Last Five Years?

**DOI:** 10.3389/fmed.2020.00487

**Published:** 2020-08-25

**Authors:** Sayam R. Dubash, Gabriele De Marco, Richard J. Wakefield, Ai Lyn Tan, Dennis McGonagle, Helena Marzo-Ortega

**Affiliations:** ^1^NIHR Leeds Biomedical Research Centre, Leeds Teaching Hospitals Trust, Leeds, United Kingdom; ^2^Leeds Institute of Rheumatic and Musculoskeletal Medicine, University of Leeds, Leeds, United Kingdom

**Keywords:** spondyloarthritis (including psoriatic arthritis), enthesitis, dactylitis, synovitis tendon inflammation, peri-tendon inflammation, tenosynovitis, ultrasonography

## Abstract

Psoriatic arthritis (PsA) is a complex heterogeneous disease with multiple inter-related pathologies such as synovitis, enthesitis, tendinopathy, and dactylitis. Clinical assessment is limited in its detail to assess pathology, thus in recent years, ultrasound (US) has become more popular, given its high sensitivity to detect inflammatory arthritis and ability to inform clinical decisions. Although a qualitative technique, US findings can be graded semi-quantitatively for grayscale (GS) and power Doppler (PD). Synovitis is frequently present in inflammatory arthritis pathologies, and in PsA, recent evidence shows a propensity for tendon and entheseal lesions. The presence of flexor tenosynovitis and flexor tendon insertional enthesopathy at accessory pulleys is supportive of the “Deep Koebner” concept. Peri-tendinous inflammation—mutual to PsA or rheumatoid arthritis (RA), is associated with soft tissue oedema with PD signal frequently at the flexor tendon compartments in PsA. Research on enthesitis in PsA/PsO has improved understanding in subclinical and clinical PsA, explored associations with progression to PsA, and investigated links to prognosis assessment. Dactylitis is a pathognomonic PsA lesion where US has enhanced knowledge of the disease course and pathology of lesions such as: flexor tenosynovitis; synovitis; and soft tissue oedema. Increased US sensitivity has also brought innovation including promising automated ultrasound scanning techniques. So, what have we learnt in recent years and what are the unmet needs to focus future research initiatives in this disabling disease? This narrative review article assesses the neoteric evidence, bringing into context the knowledge gained and highlighting potential areas of research.

## Introduction

Psoriatic arthritis (PsA) is a heterogeneous disease characterized by joint, tendon, and entheseal inflammation in both the peripheral and axial skeleton. At these sites, inflammation gives rise to pain, tenderness and swelling which is either localized around a joint or more diffuse e.g., along a whole digit (dactylitis). Categorized as one of the main disorders under the umbrella term Spondyloarthritis (SpA), PsA incorporates associated extra-articular manifestations including cutaneous psoriasis (PsO), related onychodystrophy, inflammatory bowel disease and uveitis. The musculoskeletal burden is comparable to rheumatoid arthritis (RA), with joint related damage, functional impairment and reduced quality of life over time (1).

The challenges of diagnosis in early PsA are not confined to the heterogeneity of disease, which is evident from the variety of outcome measures available (2). In contrast to RA, there are no biomarkers such as anti-citrullinated peptide antibody (ACPA) or rheumatoid factor (RF) to identify early PsA and therefore diagnosis is dependent upon identification of specific clinical features. In addition, elevation of acute phase markers such as C-reactive protein (CRP) only occurs in up to half of patients and is therefore of limited value in early PsA (3). Lastly, the absence of PsO in the presence of arthritis may lead to a label of undifferentiated arthritis. Reflecting these shortcomings, imaging has been increasingly utilized for PsA evaluation and therapy assessment.

Plain film radiographs of joints are feasible, quick to perform and low in cost, with the ability to assess progressive damage reasonably well. However, when compared to ultrasound (US) or magnetic resonance imaging (MRI), they lack sensitivity for detecting early inflammatory arthritis and associated damage (4, 5). Ultrasonography (US) has various advantages over MRI, including greater accessibility, overall reduced cost, lack of contraindications, and its availability in the clinic. However, MRI has the advantage of allowing access to sites where US has a limited acoustic window e.g., axial skeleton and all osseous based pathology. Given the mounting evidence on early treatment of active inflammation for optimal outcomes, the need for adopting sensitive imaging tools into routine practice has never been greater. The aim of this review was to evaluate the recent research literature on US use in PsA with relevance to clinical practice.

## Methods

A panel of rheumatologists undertook this project. Although no systematic search was performed as such, the Patient-Intervention-Comparison-Outcome (PICO) standard was adhered to. Intervention: the search strategy focused on scientific publications reporting on the use of US for diagnosis, management and assessment of PsA. Population: the target group included adults (≥ 18 years of age) with a diagnosis of PsA of any disease duration. Specific PsA musculoskeletal manifestations explored were synovitis, dactylitis, and enthesitis. PsA-related spondylitis was not included due to unfeasible accessibility of axial skeleton structures to US imaging techniques. Comparator: the imaging techniques appraised, whenever available, were conventional radiography, computed tomography (CT), and MRI. Outcome: no pre-set outcome measures were chosen, as the panel felt that a restricted approach would narrow the focus of the review excessively. Whenever available, measures of diagnostic accuracy (sensitivity, specificity, positive/negative predictive values, and likelihood ratios) were retrieved (Table 1).

**Table 1:** References appraised and selected for inclusion.

**Ultrasound for diagnosis**
• Synovitis Records evaluated: 80; Records included: 6 (2 relevant references from > 5 years included) • Subclinical synovitis Records evaluated: 65; Records included 4 • Tendon pathologies Records evaluated: 97; Records included 10 (of which 2 are also cited in “synovitis”) (3 relevant references from > 5 years included) • Entheseal pathology Records evaluated: 126; Records included 13 (of which 1 already cited in tendon pathologies) (1 relevant reference from > 5 years included) • Dactylitis Records evaluated: 38; Records included 5 (0 references from > 5 years) • US in differential diagnosis of PsA Records evaluated: 134; Records included 4 (of which 1 already cited in tendon) • Limitations of US in clinical practice Records evaluated: 43; Records included 1 (0 references from > 5 years) • Comparison of US with clinical examination and composite clinical scores Records evaluated: 101; Records included 5 (of which 1 is already cited in “dactylitis”); (1 relevant reference from > 5 years included).
**Ultrasound for management**
• US for management Records evaluated: 59; Records included: 2 (0 references from > 5 years) • Monitoring of PsA Records evaluated: 280; Records included 2 (0 references from > 5 years) • Remission assessment Records evaluated: 120; Records included 3 (of which 2 already cited in subclinical synovitis and comparison of US with clinical examination) (1 relevant reference from > 5 years included) • Prognosis Records evaluated: 87; Records included 4 (of which 1 already cited in tendon) • Composite US scores (joints, entheses) Records evaluated: 19; Records included 9 (of which 1 is also cited in “tendon pathologies”), (3 relevant references from > 5 years duration). • Guided injections Records evaluated: 116; Records included 4 (2 relevant references from >5 years).
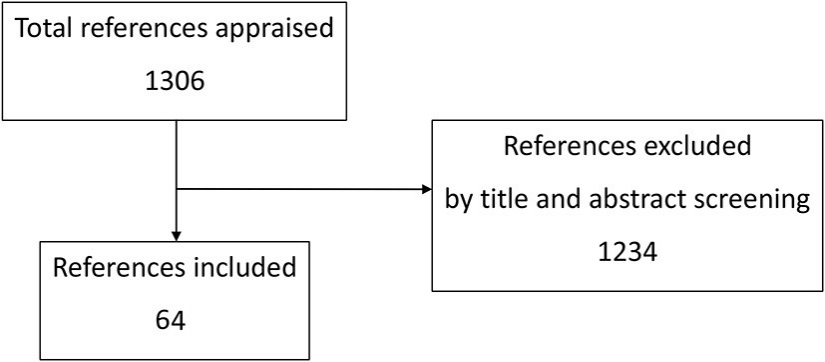

The search strategy encompassed clinical trials, well-designed cohorts and systematic reviews published in Pubmed from 2015 onwards and was performed by two members of the working party (SRD, GDM). Only publications in English were considered. Key papers before 2015 were included if considered relevant to the review.

## Results

### Ultrasound for Diagnosis

#### Synovitis

**Figure 1:**
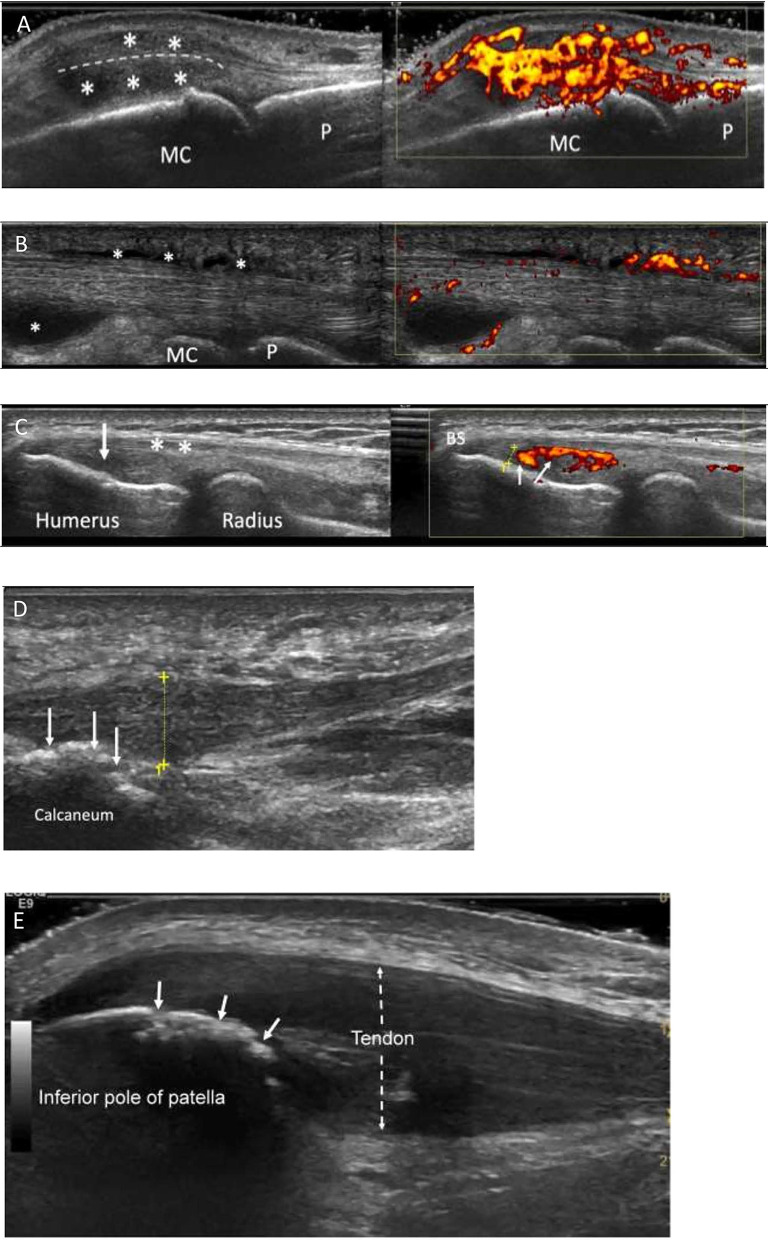
Characteristic ultrasound appearances in Psoriatic arthritis. **(A)** Longitudinal view through a metacarpophalangeal joint with synovitis. There is gray scale thickening (*between joint and extensor tendon-dotted line) and marked increased power Doppler signal (grade 3, right image) consistent with “active” synovitis and peri-tendonitis (*above tendon). MC, metacarpal; P, phalanx. **(B)** Longitudinal view through the flexor tendon on a finger. It demonstrates fluid and synovial thickening within the tendon sheath (*). There is also marked power Doppler signal within the tendon sheath (right image). MC, metacarpal; P, phalanx. **(C)** Enthesitis of common extensor origin (CEO): Longitudinal view through the common extensor origin. Hypoechogenicity (arrow/left), loss of fibrillary pattern (*), Bone spur (BS), increased Doppler signal (small arrows/right) within 2 mm (dotted line) of bone surface. **(D)** Longitudinal view through the plantar fascia of a patient with PsA. There is thickening of the fascia (dotted yellow line measuring 8.3 mm; normal < 5 mm). Power Doppler signal is rarely seen at the plantar fascia insertion. Bone irregularity is suggestive of erosive change (arrows). **(E)** Longitudinal section through the proximal patellar tendon. The tendon is markedly thickened (dotted arrow), hypoechogenic (compared to normal tendon more distally) and has lost its fibrillary pattern. The bone surface of the patella shows bone irregularity consistent with erosive change (white arrows). This was confirmed on transverse view.

Grayscale (GS) US findings in PsA are similar to those of RA in morphology with synovial hypertrophy, intra-articular effusion, enhanced power Doppler (PD) signal, and erosions (Figure 1A). However, the literature shows a trend to higher severity in RA synovitis as compared to PsA (6, 7). In these studies focussing on synovitis in PsA, US showed more prominent tendinous/entheseal involvement adjacent to synovial joints in the PsA groups (8, 9). Absence of PD signal over the hypertrophic tissues, however, did not rule out active intra-articular synovitis (6). Preliminary data suggested that the pathologic processes in the intra-articular synovia may follow –not precede- inflammation at the level of soft tissues surrounding extensor tendons in the hands (9, 10). One limitation of US, in the context of the evaluation of psoriatic polyarthritis, could be the amount of time needed to perform such investigation, as compared to clinical evaluation. However, automated US scanning techniques showed 2-fold higher sensitivity in detecting synovitis of the hands, when compared to clinical examination, and have potential for improving the current standard of care in rheumatology clinics (10).

#### Subclinical Synovitis: What Does it Mean?

Attribution of articular swelling to synovitis is better performed by US as compared to clinical examination, especially at a sub-clinical stage. Subclinical synovitis as detected by US is frequent in subjects with psoriasis and healthy individuals (up to 49.6% may show at least one abnormality, in at least one site investigated). Although less common, PD signal suggestive of subclinical synovitis was present in 24% of healthy subjects (11). It is interesting to note that in the healthy subjects recruited in this cross-sectional study, PD signal showed mild alteration (grading 1 out of 3) and lesions scoring 2 or 3 were uncommon or absent, respectively. Of note, PsA patients recruited in this study had subclinical synovitis more frequently, affecting more sites and with more severe PD signal alterations. Frequent findings of subclinical synovitis in healthy subjects (55%) and psoriatic patients (85%) were confirmed by another cross-sectional study (12). However, in this report, “active synovitis” (that is, the combination of synovial hypertrophy with PD signal) was found exclusively in psoriatic subjects (27.5%) (12). Histological evaluation would ideally be needed to disentangle the meaning of these imaging findings in pre-clinical PsA and in normal.

Subclinical synovitis -with or without subclinical enthesitis- at baseline is more frequent in psoriatic patients who develop PsA over a follow-up of 2 years (13). In PsA patients who are in clinical remission/minimal disease activity, US detection (using associated PD) of non-clinically noticeable synovitis is a predictor of short-term (6 months) flare (14).

#### Tendon Pathologies

Tendinopathic pathologies can exist in both PsA and RA but can be difficult to attribute specifically to either disease. Benjamin et al. (15) described the concept of a “functional enthesis,” an anatomical, biomechanical, and pathological feature that share fibrocartilaginous entheses proximal to regions of attachment to allow tendons or ligament to wrap around bony pulleys. It is at these sites that there is a propensity for disease in PsA which has been confirmed through US (16). Flexor tenosynovitis can be detected by high-resolution US of the hand flexor tendons as illustrated in Figure 1B. Peri-tendinous soft tissue oedema and PD signal have been reported in the 2nd−4th flexor tendon compartments of the dominant hand in one third of PsA vs. no RA patients (8, 17). Additionally, flexor tendon insertional enthesopathy occurs at accessory pulleys including new bone formation, significantly more common in PsA, supporting the “Deep Koebner” phenomenon (18). A much higher percentage of peritendinous extensor digitorum tendon inflammation was observed in PsA compared to RA (9). Soft tissue oedema was detected almost exclusively in PsA when the most clinically involved finger was assessed. Further, central slip enthesitis at the PIP joints was exclusively found in early PsA. Ultrasound detection of extra-synovial features and at the synovio-entheseal complex may be helpful in the differential diagnosis between early RA and early PsA (9).

There is some expert based consensus that the most useful anatomical sites for identifying disease at tendons (with sheaths) are at the hand flexor tendons, extensor tendon compartment of the wrist, and for peri-tendonitis (inflamed tendons without sheath) hand extensor tendons are favored over the feet extensor tendons (19).

Further recent studies have also added to the literature on significantly greater tendon sheath synovial thickening and tendon sheath PD signal observed in PsA compared to PsO without PsA (20). On a practical level, a previous study demonstrated greater peritendon extensor tendon inflammation at the MCP level in PsA than RA, indicating that it is a key characteristic of PsA, valuable in differential diagnosis (10). Importantly in PsA, the most recent evidence indicates that MCP swelling is actually attributable to not only synovitis, but also peri-tendonitis (Figure 1A), and are detectable at similar frequencies (21).

#### Entheseal Pathology

Enthesitis is considered characteristic of PsA and an early lesion throughout disease progression (22). The clinical assessment of the entheses can be impaired by lack of sensitivity and overlap with pain amplification syndromes (23, 24). Moreover, data suggest a disconnection between arthritis severity and enthesitis severity (25). Although recent studies found no correlation between the clinical and the US assessments of entheses in PsA there is potential for increased accuracy of enthesitis assessment using US scan (26, 27).

OMERACT proposed (28) a definition for the elementary lesions that characterize enthesopathy: (1) “hypoechogenicity at the enthesis”; (2) “thickened enthesis”; (3) “calcification/enthesophyte at enthesis”; (4) “erosion at enthesis”; (5) “Doppler signal at enthesis” (Figures 1C–E). Some of these lesions are structural and commonly seen in people not affected by PsA, although in PsA they tend to be more severe (29–31). Moreover, the definition of PD alterations within 2 mm from the enthesis is not accepted by the whole scientific community. Furthermore, body weight and other mechanical factors and metabolic conditions are considered confounders for enthesopathic structural lesions (17, 32). However, US-detected active enthesitis (that is, the combination of elementary lesions 1 and 5) is related to older age and higher levels of physical activity in PsA patients (33). Interestingly, inflammatory entheseal changes are commonly found in people who have psoriasis or arthralgia and psoriasis, preceding the clinical onset of PsA. In the study by Zabotti et al. (32) the presence of US evidence of enthesitis was associated with progression to PsA.

#### Dactylitis

The use of US has added to the understanding of pathologies involved in dactylitis that extend beyond the presence of synovitis and flexor tenosynovitis. In a recent study of dactylitis in PsA patients, joint synovitis was detected by US in 40% of dactylitic digits and was associated with longer duration of dactylitis and the asymptomatic “cold” type characterized by swelling but not pain or tenderness (34). Another study of psoriatic dactylitis identified PD at the accessory pulleys of affected digits, suggesting that these sites of mechanical stress may be more important in the disease process than previously thought (35). Moreover, flexor tenosynovitis is most prevalent in the majority of PsA imaged dactylitis and over half of patients also display subcutaneous oedema and synovitis (36). Unlike the OMERACT US definitions aforementioned for synovitis and enthesitis, no widely accepted ultrasound definition was present for dactylitis. Just recently, Zabotti et al. (37) have developed an US score for dactylitis, namely the DACTylitis glObal Sonographic score in PsA (DACTOS). Dactylitis elementary lesions were reviewed via a Delphi exercise of 12 experts to reach a consensus on scoring which resulted in moderate/excellent reliability for US scored lesions (37). Imaging scores of such may assist in the diagnosis and evaluation of the response of tissue compartments to therapies (38).

#### US in Differential Diagnosis in PsA

The morphological similarities between PsA synovium and that of RA leave open substantial issues related to the differential diagnosis of inflammatory arthritides. Narrowing the view on synovitis, the integration of contrast enhancement technique to US scans may assist in distinguishing across different diseases. Some evidence points to the potential of different software for quantitative analysis of the kinetic parameters of the synovial vascular perfusion pattern. In one study, this sophisticated technique has shown discriminatory ability in the assessment of RA vs. other forms of arthritis, including PsA (39). However, such advanced analysis tool would be available only in research centers for now.

Beyond synovitis, the available evidence supports the concept that PsA is mainly differentiated from RA by the involvement of non-synovial articular and peri-articular structures/tissues (17, 40). Key findings are enthesitis, peritendonitis of the extensor tendons of the hands, thickening of the pulleys of the flexor tendons of the hands, peri-tendineal dermal soft tissue oedema and bone proliferation associated with erosions (usually smaller than RA). The presence of extra-synovial features on US of the hands showed sensitivity of 68% and specificity of 88.1% for early PsA (16). Some limitations apply, since most studies were performed on limited parts of the musculoskeletal system (hands and wrists mainly). Moreover, the comparisons were made between RA, seronegative SpA and PsA (leaving out crystal-caused arthropathies, especially the chronic forms).

### The Limitations of US in Clinical Practice

On a practical level, clinical examination, which is subjective and not anatomically nor pathology specific, is complemented by the high sensitivity of US to detect inflammatory and structural lesions, clearly advantageous to identify characteristic PsA-related pathologies. Despite these significant benefits, a recent systematic review reported variable diagnostic accuracy for US in PsA, in fact confirmation of a PsA diagnosis was heavily based on clinical diagnosis and classification criteria (CASPAR) (41). One should also be cognizant that the objectivity of US is dependent upon having a skilled operator for scanning and image interpretation, and a sensitive US machine/ transducer, particularly relevant for PD signal detection. Further, it is unfeasible to scan 68 joints and numerous entheses for every patient in routine clinical practice due to time constraints, therefore comprehensive US assessments of this nature occur mostly in a research setting. There are also costs to be considered involving the purchase, running, and servicing of the equipment.

### Comparison of US With Clinical Examination and Composite Clinical Scores

A recent study has confirmed that in fact there is a significant association between clinical and US assessment of the large entheses when assessing Achilles and Patellar tendon origins (42). Furthermore, digital pain and tenderness in dactylitis was linked to US tenosynovitis GS ≥ 2 (34). However, large discrepancies have been reported between clinical examination and US findings for synovitis and enthesitis (43). In the same study, the DAPSA composite scores partially reflected Boolean's remission criteria and correlated with GS and PD synovitis but not the CPDAI (43). In another longitudinal study of 47 PsA patients, the SJC66, CRP, ESR, DAS28, and the physician global assessment were associated with PD, whereas the DAPSA was not (44). Therefore, the discordance between clinical examination and US synovitis needs further research. However, a recent report on clinical low disease activity (LDA) states, (determined by DAPSA, PASDAS, CPDAI, or MDA) suggests they are able to differentiate between high and low (MUDA) US determined disease activity (45). The unmet needs and suggested areas to focus US research in diagnosis and management of PsA are summarized in Table 2.

**Table 2:** Unmet needs in ultrasound imaging in Psoriatic arthritis.

**Topic of interest in PsA**	**Unmet needs: suggested research focus, by clinical phenotype**	
	**Psoriatic oligo/polyarthritis**	**Psoriatic dactylitis**
**Diagnosis:**		
Sensitivity and specificity:	• PsA is frequently underdiagnosed or misdiagnosed. Can US improve the PsA diagnostic yield? • The specificity of US features to PsA is an area where further knowledge may improve clinical diagnosis given that overlapping pathologies exist between diseases. • Larger sized prospective cohorts may provide further insight into the characterisation of US features useful to differentiate different inflammatory arthritides (e.g., RA, PsA/SpA, crystal arthropathies).	• Dactylitis is a unique lesion in PsA. Yet the significance of dactylitis is still unclear in terms of the overall burden of disease in PsA. Further research on this lesion and US in PsA will drive further knowledge and understanding of this pathognomonic lesion in PsA/SpA. • The nature of dactylitis from early disease onset into chronic PsA is not fully understood. More research from large PsA cohorts may explain this further. • High sensitivity US may be useful in detection of small entheseal tissue in dactylitis research.
**Synovial** Synovitis:	• Disparity between clinical examination and US findings in PsA is still not well-understood. The relationship between clinical examination and US for identifying synovitis (and enthesitis below) is poorly understood and requires further research to clarify and inform clinical practice and decision making. • The large number of joints in the Psoriatic joint clinical/US assessment is time limiting and impractical in the clinic. A condensed, time-sensitive, but valid and reliable US tool that can be easily implementable is required for practicality in clinical practice and research. • More work on longitudinal PsA/PsO cohorts needed on the subject of subclinical synovitis and its prognostic value in the short and medium term (up to 5 years of follow-up).	• What is the relationship between dactylitis and synovitis? Does this differ depending upon disease course or treatment type? • Are there any ultrasound predictors that determine why some people with dactylitis may be affected by worse outcomes? • PsA cohorts with dactylitis may provide more insight into future dactylitis research. • Is there a risk from dactylitis to overall disease related affliction and treatment in early disease? • Does synovitis represent risk of dactylitis relapse/recurrence?
**Extra-synovial** Enthesitis:	• Discordance between clinical an US enthesitis suggests further studies may inform the differentiation between PsA and non-PsA. • Is an enthesitis US composite research score needed? If so, how many entheses should be included and which ones?	• The enthesis organ concept is highly implicated in PsA, yet we do not know whether dactylitis results in more clinical/US enthesitis? • Does dactylitis represent an intermediary lesion in the disease spectrum, developing from enthesitis and next into synovitis, or could it be a more significant clinical marker of disease severity in early PsA? These are questions that future US research may be able to answer using US at time-points in PsA evolution.
Tendon related pathologies:	• Research to improve differentiation of tendon pathologies in early PsA from RA or Palindromic rheumatism is where US may inform clinical practice. • Further research is needed on imaging of tendon pulleys and sheaths in early PsA which may hold early diagnostic value. • Identification of flexor/extensor tendons, peritendinous regions: where, when, and which should we scan? Is there a significance in terms of the disease overall burden?	• Tendon pathologies are key pathological features in dactylitis and correspond to the anatomical concept of swelling across the whole digit. Further understanding of tendinopathy in this lesion may improve targeted PsA therapy per patient based upon lesion. • Flexor tenosynovitis and flexor tendon sheath and pulley pathologies are key components of dactylitis. • Peritendon inflammation: US vs. MRI, disease course and response to therapy. • Scoring is a “hot” topic: Validation of scoring methods can permit use in research and clinical settings. • Hot and cold differentiation and active pathologies: clinical examination findings could be included to encompass the best representation of dactylitis status.
**Management:** US disease activity scores/composite outcome measures for monitoring	• How should overall US disease activity be measured per patient? • Which composite measure should be used?	• Isolated US vs. combined clinical and US features of dactylitis should be considered to assess dactylitis disease activity and monitor treatment response. • Clinical trials and longitudinal cohort studies may provide further clues into this arena where the data is sparse.
Disease remission:	• Clinical vs. US remission: which should be used? How should US remission/low disease activity be defined? More research in this area is needed.	
Pragmatic, additional issues:	• Advancing technologies are emerging which allow automated and simplified assessment of joints in less time. Can they be reliably and validly implemented and with cost-effectiveness?	

## Management

### Ultrasound for Management

The EULAR recommendations for the use of imaging in the diagnosis and management of SpA in clinical practice suggest that US has a place in providing “additional information” on top of clinical examination and laboratory investigation for monitoring the activity of peripheral SpA, including PsA (46). Perhaps surprising is that in RA, recent data showed a dichotomy between the relationship of the clinical TJC and SJC with US synovitis suggesting TJC is not associated with US synovitis, but data from similarly large cohorts for PsA is missing (47). The complex relationship between clinical and US examination is poorly understood in PsA and would suggest the inclusion of US can aid the assessment of disease where clinical assessment may have missed underlying occult PsA disease activity. As well as for disease monitoring, remission status, and disease activity measures, we evaluated data for the value of US in prognostication and targeted therapeutic interventions (intra-articular injections).

#### Monitoring of PsA

One study on PsA looking at the utility of US in the clinic showed that PD signal detected at baseline was not associated with response to treatment at 4 months [either biologic or conventional synthetic disease-modifying anti-rheumatic drugs (bDMARDs, csDMARDs, respectively)] in the routine care setting (48). These findings contrast with those from studies in RA, so Højgaard et al. (48) argued that one possible explanation could be the different presentation of the two diseases (that is, RA is more symmetric/uniform than PsA). The possibility remained that PD signal grading continuum, not merely its presence, would be more relevant for prognosis. In the TICOPA imaging substudy US inflammation scores were based on summation of GS and PD and both these features were graded 0–3. In this underpowered substudy, the US-based measure demonstrated responsiveness, was aligned to clinical outcome measures at baseline, and was aligned to the change detected by clinical outcome measures between two different time-points (49).

### Remission Assessment

In PsA patients who are in clinical remission/minimal disease activity, US detection of subclinical synovitis using PD was a predictor of short-term (6 months) PsA flare (14). There is however, evidence of poor correlation between levels of clinical PsA activity (as measured by composite outcome measures) and US inflammatory findings (above all, PD signal) (50). US remission was found in 49.6% of the patients in this cross-sectional study, while clinical remission was achieved by 5.7–9.9% of the patients (depending on the composite outcome measure used). In this study, patient-reported-outcome-measures, a component of many clinical composite outcome measure, correlated with US findings worse than swollen joint count.

In another study, in 20% of PsA patients with clinically active disease (as measured by clinical composite outcome measures such as DAPSA, PASDAS, CPDAI, MDA) US assessment resulted in minimal inflammatory activity (MUDA) (43). In this study, the pain-related items, as part of the clinical composite outcome measures, were the components conditioning higher disease activity scores. The authors postulated that the evaluation of clinical treatment targets can benefit from US evaluation.

### Prognosis

In PsA patients clinically classified as oligoarthritis, US scanning has uncovered synovial hypertrophy of polyarticular distribution (51). Similar findings are relevant for the assessment of PsA prognosis. Baseline US findings of synovitis (upon GS and PD assessment) were identified as risk factors for additional articular damage in one study (52). In this study, the presence of baseline enthesopathy/enthesitis also accounted as a risk factor for articular damage.

Another study found that 26 PsA patients who were in clinical remission had low levels of PD signal on US scan. Once csDMARD or bDMARD therapy was stopped the clinical recurrence rate of PsA was high (90%). One predictor of PsA relapse was synovial hypertrophy on US scan at the time of therapy cessation (53). Further data on the assessment of remission and prognosis are expected from the UPSTREAM study (19).

### Composite US Scores

Just as there are several clinical composite scores for the assessment of PsA, US disease activity may be scored using a number of validated methods for the joints and entheses in clinical and research practice.

#### Joints

Two composite scores have been specifically developed to monitor disease activity in PsA: the 5TPD and PsA-Son composite scores with good sensitivity to detect inflammation and feasibility, but not yet validated in any other series (19).

Following the suit of many rheumatology clinical composite scores, Ficjan et al. (54) proposed two US scoring methods to assess inflammatory and structural PsA lesions, the PsA-Son13, (unilateral joints), and PsA-Son22 score (bilateral joints). They reported sufficient construct validity, reliability, and sensitivity to change for both scores. The reduced number of joints included may be considerably time saving, however there is potential to miss involved joints leading to a false reflection of overall disease activity, especially relevant for oligo/monoarticular phenotypes.

The “5 targets Power Doppler for Psoriatic disease” (5TDP) was based on joints, tendons, entheses, skin and nails scoring the highest expression of PD signal (55). The limitations were that the score does not consider multiple joint involvement from single joint involvement and may lead to under estimation of disease activity in polyarticular disease (55). A further drawback is that nail and skin US assessment is not commonplace in routine practice and therefore not practical outside of a research setting. Finally, it is notable that large joint involvement is frequent in PsA, therefore a tool initially developed for validation in RA, SOnography in LArge joints in Rheumatology (SOLAR), has been reported for its suitability for PsA (56).

#### Entheses

In a recent study, the Madrid Sonographic Enthesitis Index (MASEI), a scoring tool designed for enthesitis in SpA/PsA, failed to distinguish between enthesitis in PsA from healthy controls (57). It was found that by excluding the knee enthesis thickness and refining PD severity, marked differences could be shown between PsA patients and healthy controls, indicating that given considerable overlap of features exists between groups, setting the best discriminative thresholds for detecting pathology is imperative (57). On the contrary, a recent systematic literature review concluded that the MASEI was feasible, reliable and a valid ultrasound score for assessing enthesitis, but did not find any articles assessing MASEI as an outcome for treatment response (58). Whether clinical tenderness is derived from enthesitis or fibromyalgia can be difficult to assess, but has recently been studied using US and scored via the Glasgow enthesitis scoring system (GUESS) (59). It was found that US enthesitis was more prevalent in PsA and PsA with fibromyalgia compared to fibromyalgia alone, and clinical entheseal scores (LEI, MASES) were shown to potentially overestimate active enthesitis in fibromyalgia (59). A further preliminary enthesitis score developed in a recent GRAPPA study has reported the ability to differentiate between PsA and healthy controls (60). However, this has led to further discussion/debate on whether a further enthesitis score is actually needed, and if so, how many entheses should be included, and the suggestion that a study to prioritize differentiation of PsA from PsO and osteoarthritis/mechanical pain should be prioritized (61).

### Guided Interventions (Injections)

Ultrasound provides the ability to visualize the needle for injection procedures and therefore optimize placement accuracy. There are no specific recent studies on PsA and the effectiveness of US guided routine intra-articular injections. However, previous randomized controlled trials (RCT) in inflammatory arthritis reported significantly better accuracy of joint injection by US over the blind/palpation approach (62). In the same study, the benefit of short-term outcomes could not be demonstrated. Another larger RCT of 244 patients reported superior outcomes and cost-effectiveness with US guided injection *vs*. the conventional blind/palpation technique, with an 81% reduction in injection pain, 35% reduction in pain scores and 38% increase in responder rate (63). In contrast, a large randomized trial examining the benefit of US in a clinical tight control regimen in RA (ARCTIC) did not find any significant difference in treatment efficacy between US guided and blind/palpation guided joint injections (64). There is a clear advantage of targeting pathologically active disease through US assessment prior to US guided injection given that treatment efficacy was observed when moderate PD synovitis was present, independent of whether the joint was clinically swollen (64). Given the multiple pathologies in PsA, it would seem reasonable to study targeted US injections based on region and type of pathology. Further research may clarify whether US guided joint injection for routine intra-articular joint injections can produce superior outcomes over routine blind approach, but the most recent data is limited (6).

## Conclusions

Ultrasound is complementary to clinical examination by adding sensitivity and specificity to sites of disease in PsA enhancing the qualitative assessment. Several recent studies have shown added value of US in research by improving the understanding of disease. The clinical role of US for diagnosis is ever more assuring, yet there is discordance between clinical and US assessment that needs further research. Composite scoring measures remain research driven tools and are unlikely to be implemented in busy routine clinics in the near future. As US becomes more widely used, its function as a disease monitoring tool is promising, but further research is required to clarify its specific role in the clinic.

## Author Contributions

All authors listed have made a substantial, direct and intellectual contribution to the work, and approved it for publication.

## Conflict of Interest

The authors declare that the research was conducted in the absence of any commercial or financial relationships that could be construed as a potential conflict of interest.
